# Bundle generation for the vehicle routing problem with occasional drivers and time windows

**DOI:** 10.1007/s10696-023-09529-3

**Published:** 2024-01-09

**Authors:** Simona Mancini, Margaretha Gansterer

**Affiliations:** 1 Department of Engineering, University of Palermo, Viale delle Scienze 8, Palermo, 90128 Italy; 2 Department of Operations, Energy, and Environmental Management, University of Klagenfurt, Universitaetstrasse 65–67, 9020 Klagenfurt, Austria

**Keywords:** Routing, Occasional drivers, Time windows, Last-mile delivery

## Abstract

In this paper, we address the vehicle routing problem (VRP) with occasional drivers (ODs) and time windows (TWs). The problem (VRP-OD-TW) is an extension of the VRP-OD, where ODs serve customers within given TWs. Differently from the basic version of VRP-OD-TW, we assume that ODs not only accept single requests, but they can also serve bundles of requests. To deal with the bundle-to-driver assignment problem, an auction-based system has been designed; a company offers a set of bundles to the ODs, who bid for all the bundles they consider attractive. There is no limit on the number of bids a driver can place, but at most one bid per OD can be assigned to avoid infeasible workloads. This system could yield a large cost reduction for the company, but its success is strongly related to the bundles offered. Hence, determining bundles which are attractive for ODs and profitable for the company, becomes a crucial issue. We propose two different bundling strategies, which make use of a spatial-temporal representation of customers in a three-dimensional (3D) space. The former is based on the generation of 3D corridors, while the latter relies on 3D clustering techniques. Through extensive computational results, we show that the former technique outperforms the latter in terms of both solution quality and computational times and that both the approaches strongly outperform bundle generation techniques that neglect the temporal dimension and rely only on spatial information.

## Introduction

The key to e-commerce’s success is that customers can compare hundreds of alternatives directly from their laptop, tablet, or phone without having to visit a physical shop, and they receive purchased items directly at home, in many cases, in less than 24 h. The number of customers that systematically use e-commerce has continuously risen in the past decade; the number further swell further in the wake of the COVID-19 pandemic when physical shops remained closed for several weeks if not months (eMarketer, 2022). Obviously, the number of items purchased on a single day experiences significant fluctuations across the year. The number of orders to fulfill during a peak period (such as during Christmas or Black Friday) is significantly higher than in off-peak seasons. This may require companies to resize their fleet to fulfill such demand peaks, but hiring new drivers only for a limited period of time can be extremely costly and disadvantageous. During off-peak seasons, most of the drivers would be idle, generating inefficiencies. To overcome this issue and to reduce costs for the company, a viable solution is to onboard non-professional drivers, taken from the crowd, to fulfill orders during peak periods. This phenomenon is known as *crowdshipping*. In a "crowdshipping" system, occasional drivers (ODs) are onboarded. Such drivers can be freelance semi-professional drivers or others such as unemployed persons, part-time workers, students, or retired persons, willing to do some deliveries to increase their income. Several practical examples of successful crowdshipping exist all over the world. One of the main drawbacks of ODs is that they have not signed (and, therefore, are not bound by) any contract with the companies concerned, so they are free to accept or reject deliveries offered by the companies. This implies that, for a successful delivery system, it is crucial for these companies, to offer attractive tasks to ODs.

In Mancini and Gansterer (2022a) the authors show that by offering attractive bundles of customers to ODs, companies can achieve much higher cost reductions than if they only offer single requests or low attractive bundles. Hence, the bundle generation problem is crucial to the success of the whole system. Such issues have been studied, to the best of our knowledge, only for problems without delivery TWs for customers. However, when operating in a TW context, the bundle generation problem is supposed to be more challenging, as they might render bundles unattractive or even infeasible. Hence, new bundling strategies that take into account take customers’ spatial and temporal information are needed. We propose different innovative techniques based on a spatial-temporal customer representation to deal with problems including TWs. We show the benefit of harnessing this information, compared with the existing bundling strategies. Further, we show that having different TWs does not always imply a lower compatibility among customers; on the contrary, a large TWs difference, combined with a different spatial location, may yield to very attractive bundles if they are generated in a smart way. This finding opens the door to bundling customers who at first sight may seem not closely related.

Through an extensive computational study, we show that the newly proposed bundling approach strongly outperforms the alternative approaches for all the investigated settings.

The novelty of this paper is twofold. Firstly, we provide a new customer representation which takes into account not only the spatial location of customers but also their service TWs, representing them as points in a 3D-space, in which the third dimension represents the time component. We show the benefit of exploiting this representation by means of an extensive computational study. Secondly, we extend the corridors-based approach presented in Mancini and Gansterer (2022a) to the newly defined customers’ 3D-space. This adaptation is non-trivial, as explained in Sect. 5. Moreover, the proposed bundling approach and the spatial-temporal customer representation technique are not only specifically suited for the addressed problem, but can be exploited and generalized to a broad class of auction-based transportation problems in which delivery TWs are considered.

The paper is organized as follows. Section 2 is devoted to related literature review. A formal problem description is included in Sect. 3, while the mathematical formulation is described in Sect. 4. In Sect. 5, we detail how to construct attractive bundles of customers, and we discuss the bidding process. Computational results are reported in Sect. 6. Finally, conclusions and future developments are discussed in Sect. 7.

## Literature review

Crowdshipping is a relatively new concept; it has become an important tool to reduce last-mile delivery costs as pointed out in Archetti and Bertazzi (2020). It consists of onboarding non-professional drivers, i.e., ODs, to do the last leg of parcel distribution in e-commerce. This allows companies to efficiently cover peak periods, in which, given the huge volume of orders to fulfill, the standard owned fleet is not able to efficiently handle all requests. In other words, crowdshipping allows companies to increase transport capacity and consequently to offer high quality service to their customers (e.g., same-day delivery) for very low or no extra fees. The term crowdshipping is quite general and is used to refer to the following: (i) private drivers willing to do deliveries in their free time in order to earn an extra income (e.g., students, unemployed persons, part-time workers, and retired persons) and (ii) in-store customers who are willing to do one or more deliveries on their departing trip. In this paper, we focus on the second type of ODs.

Although crowdshipping became popular only recently, several relevant studies on it can be found in related literature. Most of these works address the topic from a managerial point of view, analyzing the potential benefit of the concept.

Le and Ukkusuri (2019) identify the relevant factors which mostly push retailers to adopt crowdshipping. An analysis of real crowdshipping systems adopted in the US is reported by Ermagun et al. (2020). The benefit of onboarding the in-store customers to deliver purchases to acquaintances and friends in their social network is discussed in Devari et al. (2017), whereas the environmental impact of crowdshipping is studied in Simoni et al. (2020). A systematic review of crowdshipping-based delivery systems can be found in Alnaggar et al. (2021).

Crowdshipping have been successfully applied as a stand-alone delivery system in several settings. Ausseil et al. (2022) analyze the case of a platform to manage dynamic delivery requests by a fleet of ODs with stochastic behavior. Customer orders and drivers willing to do deliveries dynamically join the platform. At fixed points in time, the platform collects all the unserved requests in the system and the currently available ODs. It proposes specifically suited menus of requests to each driver. The driver can select the preferred request or can refuse to serve all the proposed requests while they wait for better offers. After having received all the drivers’ selections, the platform provides a match between requests and drivers. Those drivers who have not picked any request from the menu or who have not been selected by the system can decide whether to wait for the next auction round or to leave the system. A similar setting is studied in Horner et al. (2021), where drivers can select more than one preferred requests. In Chen et al. (2018) the authors propose a delivery system in which occasional drivers may transfer goods among each others during their path. This way, a customer can be served by more occasional drivers, each one of which perform a leg of the distribution, in a multi-hop fashion. This system is particularly useful for companies operating on a large territory, or in context where occasional drivers has a limited flexibility. In Torres et al. (2022) the authors consider a stochastic problem in which the system generates potentially attractive open routes to offer to ODs. Each driver selects the most profitable route from among those available when entering the system. In Gatta et al. (2018), the authors propose a sustainable crowdshipping delivery system in which the ODs do deliveries by means of public transportation, reducing, therefore, even the number of vehicles in the network. A very similar system is addressed by Gatta et al. (2019); the difference, however, is that that ODs deliver to smart lockers instead of to private locations. Another sustainable crowdshipping system is proposed by Lin et al. (2020) in which deliveries are done on bicycles.

Such systems, which consider a set of drivers entirely composed of ODs, are specifically suited for very dynamic online environments, such as meal delivery, in which during peak hours the rejection of some orders is tolerated, as there are not enough resources to fulfill all of them. However, they are not viable for giant companies, operating in a last-mile delivery context (e.g., Amazon), for they have to reliably fulfill thousands of orders per day and are not supposed to reject any of them. In this context, the most viable option is to opt for a mixed fleet of owned vehicles and occasional drivers, as shown in Mancini and Gansterer (2022a).

In the following section, we focus on the literature on mixed-fleet crowdshipping problems, involving in-store customers. Archetti et al. (2016) are the first to introduce the vehicle routing problem with ODs (VRP-ODs). The authors assume that at the most one customer can be assigned to an OD (i.e., bundling is not allowed) and that the compensation is fixed and does not depend on the detour that the OD concerned must take. Hence, drivers differ among each other only by their location, but their behavior is not modeled, and they cannot reject assignments but are supposed to accept all requests which require a detour below a certain threshold. It is assumed that the threshold and compensation payment are equal for all ODs. More recently, an algorithm to solve this problem, based on the integration of Variable Neighborhood Search and of Machine Learning has been presented in Di Puglia Pugliese et al. (2022a).

Macrina et al. (2017) and Di Puglia Pugliese et al. (2022b) address an extension of the work by Archetti et al. (2016). The authors consider customers’ delivery time windows and allow ODs to serve multiple customers during their detour. However, as in Archetti et al. (2016) they do not consider an auction system but assume that drivers would accept all the assignments that require a detour shorter than a threshold and consider fixed compensation. Therefore, they neglect features such as the ODs’ flexibility or their willingness to do deliveries. For this reason, our approach is not directly comparable to theirs. In fact, the previous works are based on the assumption that ODs would automatically accept the compensation proposed by the company. We contribute to existing studies by covering the fact that not all ODs are willing to perform a delivery for exactly the same price.

In Macrina et al. (2020) the authors keep the same assumptions but consider transshipment nodes where ODs can collect parcels to be delivered from the company-owned fleet. Hence, a two-echelons distribution system is investigated. In Yu et al. (2021) the authors also consider a two-echelons system, but they allow customers to be served either with home delivery or to shared delivery locations, called covering points. While the owned fleet can operate in both modes, ODs can only perform home deliveries. A very similar setting is addressed in Yu et al. (2022), in which customers can choose among the two options (home delivery or delivery to alternative delivery point) or can let the company choosing for them.

Arslan et al. (2019) address a dynamic pickup-and-delivery system, involving both the company’s fleet and a set of ODs. They consider OD’s flexibility through a set of parameters such as maximum detour and maximum number of stops accepted. However, those parameters are taken into account only to evaluate customer-driver compatibility, and they do not influence the compensation. These are computed as a fixed fee for each delivery task plus a per-mile component which is multiplied with the detour. A pickup and delivery system with transshipment nodes has been presented in Voigt and Kuhn (2022), whereas in Yu et al. (2023) simultaneous pick up and deliveries are allowed.

Behrend and Meisel (2018) also consider the ODs’ flexibility. However, similar to Arslan et al. (2019), this flexibility is assumed to determine the set of feasible customer tuples to be assigned to ODs, and no bidding system is imposed on them.

Dayarian and Savelsbergh (2020) address a stochastic and dynamic problem, involving in-store customers as ODs where their arrivals and their delivery capacity is taken as unknown. Compensations are considered fixed, but different compensation levels are tested, and their impact on the usage of ODs is analyzed.

In Kafle et al. (2017) a mixed delivery system is investigated, where cyclists and pedestrians are integrated with the owned fleet. Crowdshippers submit bids which can be either accepted or rejected by the company. Those that are selected for delivery meet the trucks at transshipment locations where they collect the parcels to be delivered.

Dahle et al. (2019) are the first to consider the possibility of rejecting assignments if the compensation offered is too low. This issue is, however, not addressed within a bidding system in which drivers submit acceptable prices but assumed to be a minimum compensation threshold for each OD. This threshold can differ among ODs, but compensations are defined a priori by the company. The ODs’ willingness to work is not taken into account. Boysen et al. (2022), do not consider in-store customers as ODs but propose to exploit distribution centers’ employees for crowdshipping online orders on their way back from work. These employees are supposed to communicate a minimum expected earning per time unit as well as a maximum acceptable driving time. The company decides which tasks to assign to each employee to maximize the number of tasks carried out by the employees. Compensation is decided by the company. What emerges from their computational experiments is that onboarding ODs to serve customers far from the distribution center would yield a clear advantage for the company; on the contrary, it would be cheaper to serve nearer customers with company-owned fleet. These results coincide with the findings of Mancini and Gansterer (2022a), showing the clear benefit of opting for a mixed delivery system (i.e., combining owned fleets with ODs). Mancini and Gansterer (2022a) analyze an auction-based system in which the company a priori generates attractive bundles of requests without having any information on available ODs. This study is the first to consider ODs’ flexibility as well as their willingness to accept bundles of requests. Both are assumed to be the parameters that influence the compensation requested by the driver to serve a specific bundle. Flexibility represents the maximum detour acceptable for a driver. This value is not related to the compensation received but only to the amount of time for which the driver is available. Willingness is a measure of how much the driver is willing to win the auction and get the job. The higher her willingness, the more competitive the price offered in her bid. Given the detour needed and the number of stops required, it is possible to derive a “neutral bid”, which corresponds to the correct price to offer for that bundle to stay in the market. A neutral bidder is characterized by a willingness $\Phi _{k} = 1$, where $\Phi _k$ is a multiplier of the correct price. Values of $\Phi _k$ lower than 1 indicate a high willingness to work. In fact, in this case, the driver is willing to perform the delivery for a price more competitive with respect to the market in order to increase her chance to get the job. Conversely, values greater than 1 indicate a low willingness to work. In fact, this means that the driver is willing to perform the delivery only if she receives a very good compensation, higher than the market price. These two features are not correlated among each other. There could be a driver willing to perform only small detours (low flexibility), but at a very competitive price (high willingness). Also, there could be a driver with a lot of spare time, who is willing to make even long detours (high flexibility), but only if the compensation paid is very high (low willingness).

Although offering bundles of requests has become a common practice in collaborative logistics, this aspect has received scant attention in the last-mile delivery context.

The advantage of offering bundles of orders, in an auction system, instead of single requests, has been discussed in detail in Gansterer and Hartl (2018a). This is based on the concept of *subadditivity* of costs, i.e., the fact that serving a bundle of requests might require a lower total cost than the sum of the costs of serving all requests individually. The advantages of bundling requests have been discussed in different contexts, such as the cooperative liner shipping network design, (Buer and Haass 2018) and collaborative logistics in a dynamic environment (Wang and Kopfer 2015).

To highlight the contribution of our work to the existing literature, we list in Table 1 the features discussed above. As can be seen in the table, our study clearly fills a gap in the existing literature. To the best of our knowledge, we are the first to consider an auction-based system, including a bundling and bidding phase, drivers’ flexibility, and their willingness to work for the VRP-OD-TW. It should be noted that even if delivery TWs have been already addressed in the crowdshipping-related literature, the problem of generating bundles of customers for addressing the problems with TWs is still a mostly unexplored area in the field.

**Table 1:** Overview of features addressed in the literature

	Mult. cust	Compens	Flexibility	Willingn	Bundl	Bids	Mixed fleet	TWs
Archetti et al. (2016)	No	No	No	No	No	No	Yes	No
Kafle et al. (2017)	Yes	Yes	Yes	Yes	No	Yes	Yes	No
Macrina et al. (2017)	Yes	Yes	No	No	No	No	Yes	Yes
Behrend and Meisel (2018)	Yes	Yes	Yes	Yes	No	No	No	No
Arslan et al. (2019)	Yes	Yes	Yes	No	No	No	No	Yes
Dahle et al. (2019)	Yes	Yes	Yes	Yes	No	No	Yes	Yes
Dayarian and Savelsbergh (2020)	Yes	Yes	Yes	No	No	No	No	Yes
Macrina et al. (2020)	Yes	Yes	No	No	No	No	Yes	Yes
Ausseil et al. (2022)	No	Yes	Yes	Yes	No	No	No	Yes
Boysen et al. (2022)	Yes	Yes	Yes	Yes	No	No	No	No
Mancini and Gansterer (2022a)	Yes	Yes	Yes	Yes	Yes	Yes	Yes	No
Torres et al. (2022)	Yes	Yes	Yes	Yes	Yes	No	Yes	No
Voigt and Kuhn (2022)	Yes	Yes	Yes	No	No	No	No	No
Yu et al. (2023)	No	Yes	Yes	No	No	No	Yes	No
**Our paper**	**Yes**	**Yes**	**Yes**	**Yes**	**Yes**	**Yes**	**Yes**	**Yes**

## Problem description

In this section, we describe the VRP-ODs-TWs approach. A company has to serve a set of customers (*I*); for each of these locations, the demand $q_i$ expressed in volume units, and the delivery TW, $[e_i,l_i]$, is known. We suppose that all deliveries start from a single depot (0). Hence, the set of nodes involved in the network can be expressed as the union of the customers and depot 0, as $I_0=I \cup 0$. For each pair of nodes (*i* and *j*) in $I_0$, the travel distance $d_{ij}$ and travel cost $c_{ij}$ are known. Further, for each customer, the company can choose between three options: (i) serving the customer with its own fleet, (ii) assigning it to an OD who will perform the delivery, or (iii) organizing a direct shipment, using a taxi service for a very high price. The third option is not profitable for the company and is chosen only when it is not possible to fulfill all the requests with the the first two options. This third option is introduced to ensure feasibility of all the instances.

The owned fleet is constituted by a set of *M* identical vehicles with a loading capacity of $Q_{max}$. Each vehicle in the owned fleet starts from the depot after $e_0$ and must return to it before $l_0$, where $[e_0,l_0]$ is the TW for the depot. A set of ODs ($\Omega$) is available. We assume that the ODs start from the depot and do a set of deliveries on their way back home (or more generally, on the way to their final destinations). The capacity of an OD’s vehicle, indicated by $Q^{OD}_{max}$, is considered homogeneous among all ODs and is computed by considering the loading capacity of a medium-sized car. Therefore, we assume $Q^{OD}_{max} < Q_{max}$.

The company receives a set *K* of bids from the ODs. For each bid, the bundle of customers with which it is associated ($\tau _k$), the bid $b_k$, and the driver who placed the bid, $o_k$ are known. We assume that each OD can submit bids for an unlimited number of bundles, and at most, one bid per OD can be accepted by the company. This allows to avoid the problem of overexposure of bidders by preventing situations in which the same OD wins several bundles but does not have the capacity to serve all them, which is a relevant issue in the auction theory (e.g., Englmaier et al. 2009). This assumption is very common in the literature and it is present in several studies, such as Gansterer and Hartl (2018a) and Gansterer et al. (2019), dealing with auction-based mechanisms in collaborative transportation.

If a bid is accepted, all customers associated with it are assigned to the corresponding OD. As stated above, a customer can be assigned to at the most one driver. This implies that all the bundles selected to be assigned to ODs are disjoint. The goal of the problem is to minimize the total cost given by the sum of the routing costs for the owned fleet, the costs associated with the accepted bids and the cost for fulfilling, by a taxi service, the unserved orders.

To summarize, the whole framework consists of the following three steps:

1. The company generates potentially attractive bundles without having any information about the ODs who are willing to join the system.
2. ODs place bids on the bundles they find attractive. The bids are based on ODs’ location, their time availability, their willingness to work, their flexibility in accepting detours, and their tolerance regarding extra waiting time for meeting TWs.
3. After the company has received all the bids, it decides which bids to accept, which customers to serve with the owned fleet, and which to serve by a costly direct shipment, using a taxi service. Finally, the routing plan for the owned fleet is generated.

The overall optimization problem faced by the company is presented in the section that follows.

## Mathematical model

For the mathematical model, the following input data as well as decision variables are used:

**Table:** 

	Input data
*I*	Set of customers
$I_0$	Set of nodes involved in the network (customers plus depot)
$\Omega$	Set of ODs
*K*	Set of bids
*M*	Number of available vehicles
$\rho$	Cost of serving a customer by a direct shipment with a taxi service
$c_{ij}$	Travel cost between node *i* and node *j*
$q_i$	Demand of customer *i*
$[e_i,l_i]$	Delivery TW for customer *i* (depot TW if $i=0$)
$s_i$	Service time for node *i* (customer or depot nodes)
$Q_{max}$	Capacity of owned-fleet vehicles
$Q^{OD}_{max}$	Capacity of ODs’ vehicles
$b_k$	Price of bid *k* offered by an OD
$\tau _k$	Bundle related to bid *k*
$o_k$	OD who submitted bid *k*
$C_k$	Set of customers belonging to bundle $\tau _k$

**Table:** 

	Decision variables
$Z_{i}$	Binary variable taking value 1 if customer *i* is visited by a company owned vehicle and 0 otherwise
$X_{ij}$	Binary variable taking value 1 if node *j* is visited by a company owned vehicle just after node *i* and 0 otherwise
$Y_{k}$	Binary variable taking value 1 if bid *k* is accepted and 0 otherwise
$U_{i}$	Binary variable taking value 1 if customer *i* is served with a direct shipment by a taxi service
$Q_i$	Non-negative variables representing the cumulative load at node *i*, expressed as the total quantity of demand delivered by a vehicle along its route, when leaving node *i*
$T_i$	Non-negative variables representing the arrival time at node *i*
$L_{ij}$	Load carried on arc (i,j)

The VRP-OD-TW can be modeled as follows:

1 $$\begin{aligned}{} & {} \min \sum _{i \in I_0} \sum _{j \in I_0}c_{ij}X_{ij}+\sum _{k \in K} b_kY_k+\rho \sum _{i \in I}U_i \end{aligned}$$

2 $$\begin{aligned}{} & {} \sum _{j \in I}X_{0j} \le M \end{aligned}$$

3 $$\begin{aligned}{} & {} Z_j + U_j + \sum _{k \in K |j \in C_k }Y_k = 1 \;\;\;\;\;\; \forall j \in I \end{aligned}$$

4 $$\begin{aligned}{} & {} \sum _{k \in K |o_k=\omega }Y_k \le 1 \;\;\;\;\;\; \forall \omega \in \Omega \end{aligned}$$

5 $$\begin{aligned}{} & {} {{\sum _{i \in I_0}X_{ij} = Z_j \;\;\;\;\;\; \forall j \in I}} \end{aligned}$$

6 $$\begin{aligned}{} & {} {{\sum _{i \in I_0}X_{ij} = \sum _{i \in I_0}X_{ji} \;\;\;\;\;\; \forall j \in I }} \end{aligned}$$

7 $$\begin{aligned}{} & {} Q_j \ge Q_i+q_j-2Q_{max}(1-X_{ij}) \;\;\;\;\;\; \forall i \in I \;\; \forall j \in I \end{aligned}$$

8 $$\begin{aligned}{} & {} 0 \le Q_j \le Q_{max} \;\;\;\;\;\; \forall j \in I \end{aligned}$$

9 $$\begin{aligned}{} & {} \sum _{j \in I_0} L_{ji} - \sum _{j \in I_0}L_{ij}=q_iZ_i \;\;\;\;\;\; \forall i \in I \end{aligned}$$

10 $$\begin{aligned}{} & {} \sum _{j \in I_0} L_{j0} - \sum _{j \in I_0}L_{0j}=-\sum _{j \in I}q_jZ_j \end{aligned}$$

11 $$\begin{aligned}{} & {} {{ L_{ij} \le Q_{max}X_{ij} \;\;\;\;\;\; \forall i \in I_0 \;\; \forall j \in I_0 }} \end{aligned}$$

12 $$\begin{aligned}{} & {} L_{i0} = 0 \;\;\;\;\;\; \forall i \in I_0 \end{aligned}$$

13 $$\begin{aligned}{} & {} T_j \ge T_i + t_{ij} + s_i -l_0(1-X_{ij}) \;\;\;\;\;\; \forall j \in I \;\;\; \forall i \in I_0 \end{aligned}$$

14 $$\begin{aligned}{} & {} T_j \le l_0-s_j-t_{j0} \;\;\;\;\;\; \forall j \in I \end{aligned}$$

15 $$\begin{aligned}{} & {} e_j \le T_j \le l_j \;\;\;\;\;\; \forall j \in I \end{aligned}$$

16 $$\begin{aligned}{} & {} T_0 \ge e_0 \;\;\;\;\;\; \forall j \in I \end{aligned}$$

17 $$\begin{aligned}{} & {} {{ X_{ij} \in \{0,1\} \;\;\;\;\;\; \forall i \in I_0 \;\; \forall j \in I_0}} \end{aligned}$$

18 $$\begin{aligned}{} & {} {{Y_{k} \in \{0,1\} \;\;\;\;\;\; \forall k \in K }} \end{aligned}$$

19 $$\begin{aligned}{} & {} {{Z_{i} \in \{0,1\} \;\;\;\;\;\; \forall i \in I}} \end{aligned}$$

20 $$\begin{aligned}{} & {} {{U_{i} \in \{0,1\} \;\;\;\;\;\; \forall i \in I}} \end{aligned}$$

The objective function is reported in (1). It minimizes the total costs for the company, which are composed of the following: (i) owned fleet routing costs, (ii) compensation paid for the accepted OD bid, and (iii) costs related to direct shipments by taxi service. The company cannot use more vehicles than those available in the owned fleet, as imposed by Constraints (2). Constraints (3) ensure that each customer is served by the company, a direct shipment by taxi service, or assigned to one of the ODs. At most one bid for each OD can be accepted, which is ensured by Constraints (4). If a customer is served by the owned fleet, it must be visited only once, as stated in Constraints (5). Constraints (6) guarantee route continuity. Constraints (7) play a double role: tracking cumulative load at nodes and ensuring sub-tour elimination, while Constraints (8) guarantee that vehicle capacity is respected. Constraints (9)–(12) are not explicitly needed by the model, but act as valid inequalities and help to strongly reduce computational times, as pointed out in Mancini and Gansterer (2022a), where the authors show that computational times can be reduced by more than 700 times.

Constraints (9) impose that the quantity delivered to each customer must be equal to its demand. Constraints (10) ensure that the total delivered quantity is equal to the sum of the demands of the customers served by the company’s fleet. Constraints (11) limit, for all the arcs, the load carried by a vehicle to its capacity $Q_{max}$. Constraints (12) force the vehicles to return empty to the depot. Arrival time at nodes is tracked by Constraints (13), while Constraints (14) force the vehicles to return to the depot before the end of the depot TW. Constraints (15) guarantee that customer delivery TWs are respected, while Constraints (15) forbid the vehicle to leave the depot before its opening TW started. Finally, Constraints (17)–(20) specify variable domains.

## Bundle generation and bidding

Attractive bundles generation has already been investigated in the field of collaborative logistics, where two or more carriers establish a coalition, according to which, they exchange customers with their partners to maximize the profits of all the participants in the coalition (Gansterer and Hartl, 2018b). Those collaborations are typically assumed to be managed by a central platform that receives information about the requests to offer to the coalition and runs an auction to assign them most profitably. The literature refers to *centralized collaborations* if the platform has perfect information about the carriers’ situations including existing customers and relevant costs. In *Decentralized collaborations* it is assumed that carriers share only partial information and trade only a subset of the requests on the platform. Bundles of requests can be generated either by the platform (e.g., Gansterer and Hartl, 2016) or by the carriers who are bidding to obtain some additional customers (e.g., Berger and Bierwirth, 2010). Offering all the combinations of requests as bundles is not a viable approach, since the number of generated bundles increases exponentially with the number of requests, making the problem intractable even for a limited number of requests. Therefore, the platform can typically generate only a limited subset of the bundles (Gansterer and Hartl, 2018a). It is of crucial importance to generate potentially attractive bundles, as a poor selection of the offered bundles would probably yield a low gained profit for the participants. In Mancini and Gansterer (2022a) the authors present innovative strategies for generating profitable bundles in an auctions-based system if no TWs have to be taken into account.

In these settings, bundles can be generated by making use of only spatial information. However, if TW requirements do exist, neglecting the temporal aspects might yield very poor or even infeasible, solutions. Customers with short distances among them but being associated with rather different TWs can be considered a profitable bundle by classical spatial bundling methods, but they are actually very unattractive, as serving them in a row could imply very long waiting times and/or very long detours.

**Fig. 1:**
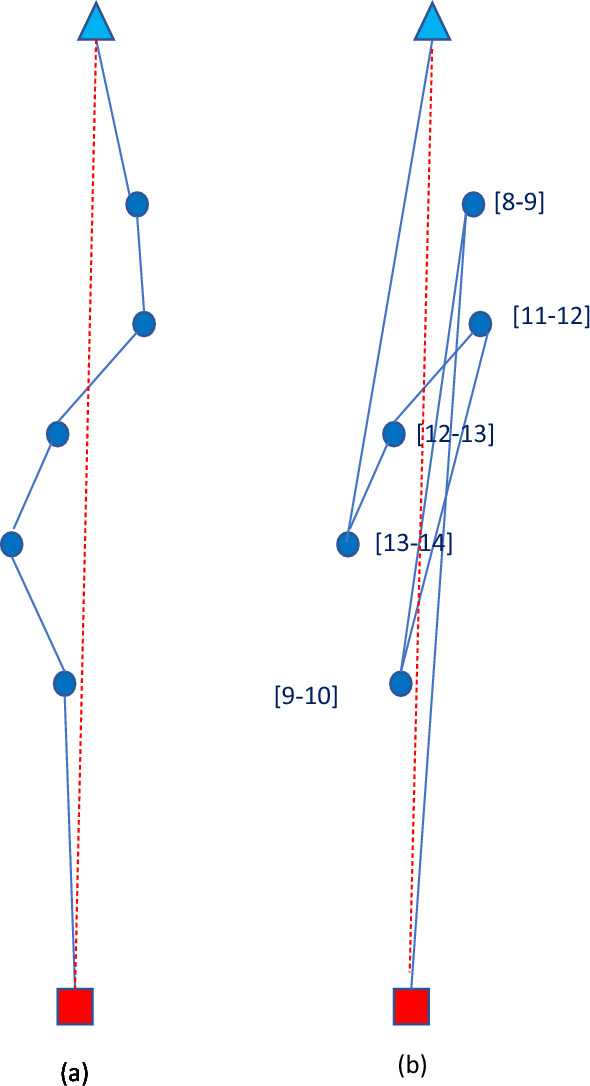
Representation of a bundle which is very attractive if no TWs are considered (**a**), but becomes very unattractive if we consider TWs (**b**)

In Fig. 1, we depict a bundle which seems very attractive for a driver if the temporal component is ignored, while it becomes very unattractive if we consider TWs. This is so because of the long detour required. The depot is represented as a red square, customers as blue circles, and the OD’s final destination as a light-blue triangle.

### Customer representation

**Fig. 2:**
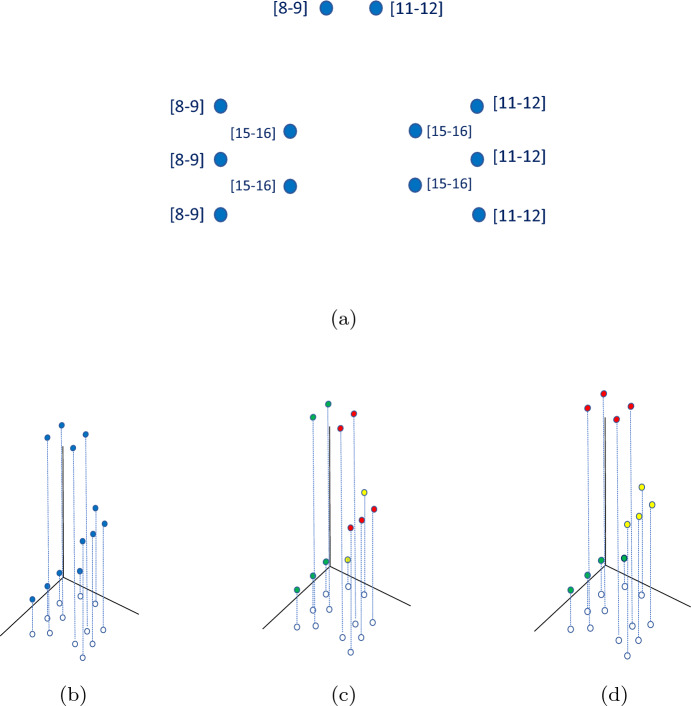
An example of bundle generation with and without considering the temporal dimension. Subfigure (**a**) reports customers’ locations and TWs, (**b**) depicts customers’ 3D representation, (**c**) shows the bundles of customers generated neglecting TWs, and in (**d**) bundles obtained considering the temporal aspect can be seen. The requests belonging to the same bundle are depicted in the same color

To locate each customer in a 3D space, we use the following representation: Each customer *i* is identified by a triplet composed of the spatial coordinates of the location and the middle point of the delivery TW $({\hat{x}}_i,{\hat{y}}_i,m_i)$, where $m_i=e_i+(l_i-e_i)/2$. By making use of this structure, each customer can be represented as a 3D-point, where *x* and *y* coordinates correspond to the actual spatial coordinates, while the third dimension corresponds to $m_i$. The distance between customers is then computed as the euclidean 3D distance between the corresponding points. This metric allows us to consider not only the similarity of locations but also the similarity of TWs. Two customers who are very close in space but have completely different TWs would be seen as very distant according to this metric. In Fig. 2, we depict a set of customers to be served as well as their 3D representation. Further, we show the optimal solution if they are split into three clusters (bundles) per their spatial location (2D representation) and 3D representation, considering TWs. It can be seen that the 2D representation generates unprofitable clustering, for a driver willing to serve the green or red bundle has to serve some customers in the bundle and then wait several hours to serve the remaining ones. Such bundles would be time–costly and therefore unattractive for drivers. Also, the yellow bundle contains two customers who are very near in space but very far in time. Conversely, all the bundles, generated by considering a 3D representation, are considerably more attractive, as the customers belonging to the same bundle are similar both in the spatial and temporal dimensions.

As discussed above, to create attractive bundles, the temporal component cannot be neglected. In this study we propose and compare two different 3D bundle generation strategies, both benefiting from the information about customers’ TWs. The first one, named *Clustering-3D*, aims to find clusters of customers maximizing the intra-cluster similarity. The second one, denoted as *Corridors-3D approach*, explores 3D corridors in a combined spatial-temporal space. It bundles customers belonging to the same corridor.

### The clustering-3D approach

The idea behind this approach is to create spatial and temporal clusters of customers. More precisely, in the 3D virtual environment, we add time as a third dimension in addition to spatial coordinates. The bundles of customers obtained in this way are potentially more profitable and more attractive for ODs, as they group together *similar* customers, i.e., customers who are relatively near each other in both spatial and temporal dimensions. Such bundles are assumed to be profitable because a single driver can serve all the customers in the bundle with relatively small detours and low waiting time. Customers who are very near in space but with very different TWs may require both long detours given the zig-zag paths to meet TWs and longer waiting times because of which the driver has to wait for the opening of the delivery TW of the next customer. These two aspects clearly have a very negative impact on the attractiveness of the bundles, and even assuming that there are some drivers willing to serve them, the compensation price, i.e., the bid, demanded by the OD would be rather high and, hence, not profitable for the company.

Different metrics can be used to create clusters of 3D items (and generally in clusters of items with *k* features), which can be represented in a *k*-dimensional space (Assent, 2012). However, one of the most prominent techniques for 3D data with numerical features is to represent these features in a 3D space, calculate distances among items by using the 3D Euclidean distance formula, and generate clusters that minimize the intra-cluster average distance. We follow this approach, but instead of using classical clustering techniques such as the $k-means$ algorithm, we propose a MIP formulation, which creates $N_n$ clusters, minimizing the average intra-cluster distances, assuming that the sum of the demands associated with each cluster does not exceed the ODs’ trunk capacity. The distance between two customers, *A* and *B*, is computed as the 3D Euclidean distance in the spatial-temporal 3D space, $d_{AB}=\sqrt{(x_A-x_B)^2+(y_A-y_B)^2+(m_A-m_B)^2}$. The intra-cluster distance is identified, for each cluster, as the largest distance between the two customers belonging to the cluster.

The MIP model involves the following additional sets and decision variables:

**Table:** 

$N=\{1..N_n\}$	Set of clusters
$w_{in}$	Binary variable having value 1 if customer *i* is assigned to cluster *n* and 0 otherwise
$R_n$	Non-negative variable representing the intra-cluster distance for cluster *n*

The problem can be formulated as follows:

21 $$\begin{aligned} \min \sum _{n \in N}R_n/N_n \end{aligned}$$

s.t.

22 $$\begin{aligned}{} & {} \sum _{i \in I} w_{in} \ge 1 \;\;\;\; \forall n \in N \end{aligned}$$

23 $$\begin{aligned}{} & {} \sum _{n \in N}w_{in}=1 \;\;\;\; \forall i \in I \end{aligned}$$

24 $$\begin{aligned}{} & {} R_n \ge d_{ij}(w_{in}+w_{jn}-1) \;\;\;\; \forall i \in I \;\; \forall j \in I \;\; \forall n \in N \end{aligned}$$

25 $$\begin{aligned}{} & {} \sum _{i \in I}q_i w_{in} \le Q^{OD}_{max} \;\;\;\; \forall n \in N \end{aligned}$$

The objective of the problem is to minimize the average intra-cluster distance as indicated in (21). The constraints (22) ensure that each cluster contains at least one customer, and, consequently, that exactly $N_n$ clusters are created. These constraints (23) force each customer to be assigned to exactly one cluster. The intra-cluster distance for each cluster is computed by means of constraints (24). Finally, the constraints (25) ensure that the total demand of customers within a cluster does not exceed the maximum allowed number.

A lower bound on the minimum number of clusters $N_{min}$ is necessary to create a feasible partition of customers without exceeding capacity constraints, and this can be computed as $N_{min}=\lceil \sum _{i \in I}q_i/Q^{OD}_{max} \rceil$.

To generate potentially attractive bundles, we run the above described mathematical model for different values of $N_n$ varying in the range $[N_{min},|I|]$. Considering $N_n=|I|$ corresponds to force the model to generate exactly |*I*| bundles, each one of which contains a single customer. The set of bundles offered in the auction includes all the clusters generated with the different values of $N_n$, excluding duplicates.

It is worth mentioning that even if we choose the 3D-Euclidean distance in the spatial-temporal space, different metrics can be easily adopted simply by modifying the rule based on which we compute $d_{AB}$.

### The corridors-3D approach

While in clustering algorithms the basic common idea is to minimize the intra-cluster distance, notwithstanding the metric used to compute such a distance, in the Corridors-3D approach, we use another kind of similarity, aiming at grouping together customers who are not necessarily close to each other but can be conveniently covered by an OD. Let us assume two customers, *A* and *B*, who are two hours of travel apart and have identical TWs [8–9]. There are two other customers *C* and *D*, also two hours of travel apart from each other, but they have TWs [8–9] and [10–11], respectively. Whatever metric we use to compute the distance, the bundle *AB* would always be the preferred one. This would cause the creation of very poor bundles, for *AB* are incompatible, as their TWs do not allow them to be served by the same driver. Conversely, the different TWs between *C* and *D* have a positive benefit, i.e., they can be served by the same driver without requiring any additional waiting time. Hence, *C* and *D* are very good candidates to become, or to be part of, an attractive bundle. The large travel time required to reach *D* from *C* allows to arrive at *D* exactly on time for the TW opening, erasing the negative effect of the temporal distance. With the Corridors-3D approach, we are the first to take into account the possible positive effect of temporal distance among customers.

The method is based on the same general concept of corridors proposed by Mancini and Gansterer (2022a) in which the customers are split in equally isized circular corridors, starting from a depot and representing potential paths to reach ODs’ final destinations. In Mancini and Gansterer (2022a), as customers are not associated with delivery TWs, only the spatial component is considered to generate these corridors, which results in planar (2D) corridors falling in the customers area. Given that, in our case, the customers are associated with TWs, using the bundles generated by the method proposed in Mancini and Gansterer (2022a) could yield to very poor bundles, for TWs may impose a zig-zag path. This might result in long detours even for the the shortest path as depicted in Fig. 1. To overcome this issue, we generate 3D corridors in the spatial-temporal space, taking into account that the vehicle is moving at a given speed *v*. The corridors are not located anymore on the horizontal plane but on a plane named *P*. The inclination of *P* depends on the vehicle speed. The faster the vehicle, the steeper the plane (i.e., the larger the angle formed with the horizontal plane). If we ideally draw a circle of radius *r* around the depot, lying on plane *P*, all the points included in this circle are reachable within a time *r* by a vehicle starting at time *t*0 from the depot with *t*0 being the height of the intersection point between the *P* and the vertical line passing the depot. We identify this intersection point as the origin of the associated plane. If we consider two parallel planes, associated with two different origins, i.e., *t*0 and *t*1, then the cylinder generated by connecting these two planes represents all the points reachable, within a time *r*, starting between *t*0 and *t*1 from the depot.

The method can be analytically described as follows:

The Corridors-3D method requires to identify a circular sector centered at the depot, defined by the smallest angle $\alpha$ for which all the customers lie in this sector. The identified circular sector is then split in $n_s$ homogeneous sectors, each one associated with an angle of $\alpha \ n_S$, (see Fig. 4).

**Fig. 3:**
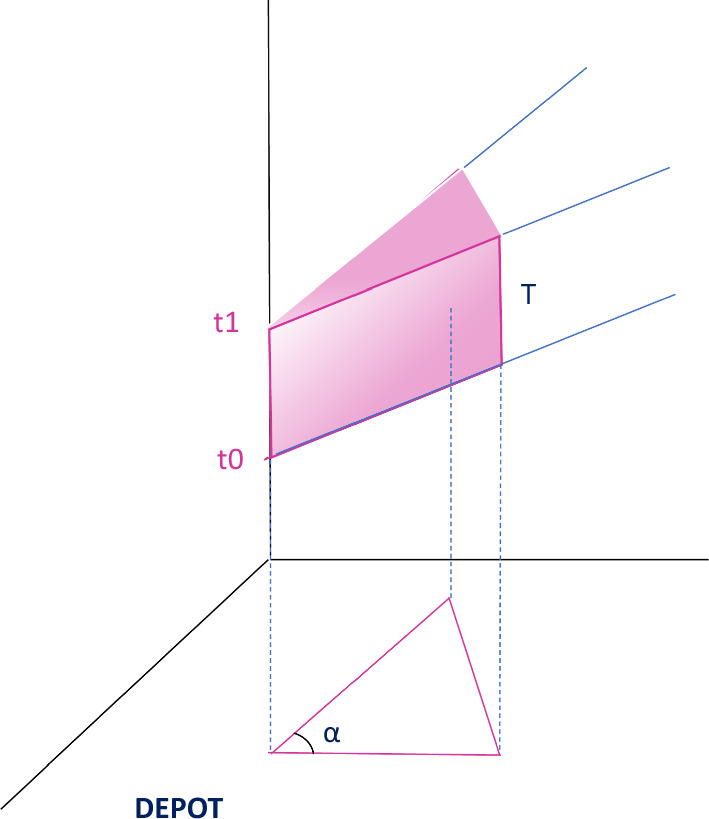
A 3D-corridor in a 3D spatial-temporal space

The sector is then projected on a plane *P*0, having as origin *t*0 and on a plane *P*1, having as origin *t*1 such that $t1-t0=T$. *T* is an input parameter for the algorithm. The sector is split into $n_s$ identical sectors and named corridors and defined by an angle of size $\alpha /n_s$. Each corridor on plane *P*0 is then connected to the corresponding corridor on plane *P*1, creating a set of 3D slices. A graphical representation of one of these slices is given in Fig. 3. All the slices are sequentially explored, and all the customers, belonging to the same slice are grouped together in a bundle. From a more operational point of view, given a circular sector $n_s$ and two time instants, namely $t_0$ and $t_1$, the following procedure is exploited to generate bundles. We connect with a segment every customer to the depot and calculate the angular coefficient of this segment, named $a_i$. We then identify all the customers for which $\alpha + (n_s-1)alpha \le a_i \le$ $\alpha + (n_s)alpha$. We call this set $S_{n_s}.$ We then construct the slice related to $t_0$ and $t_1$, drawing the two planes, $P^0$ and $P^1$, intersecting the *z* axis on $t_0$ and $t_1$, respectively. We then consider the 3D-circular sector, named slice, identified by these two planes, (see Fig. 1). Then, we examine all the points associated with the customers in $S_{n_s}$, i.e., all the points having the same x and y coordinate of the customer and the z coordinate corresponding to the starting of the TW associated with the customer, and we create a subset of $S_{n_s}$, named $S'_{n_s}$, in which we insert only customers for which the correspondent point belongs to the slice. If the sum of the demands of customers in $S'_{n_s}$ is lower than the capacity of an OD vehicle, $Q^{OD}_{max}$, we generate a bundle containing all of them. In cases where the total demand of these customers exceeds the vehicle capacity ($Q^{OD}_{max}$), the clustering-based approach is run on this subset of customers to generate feasible bundles.

**Fig. 4:**
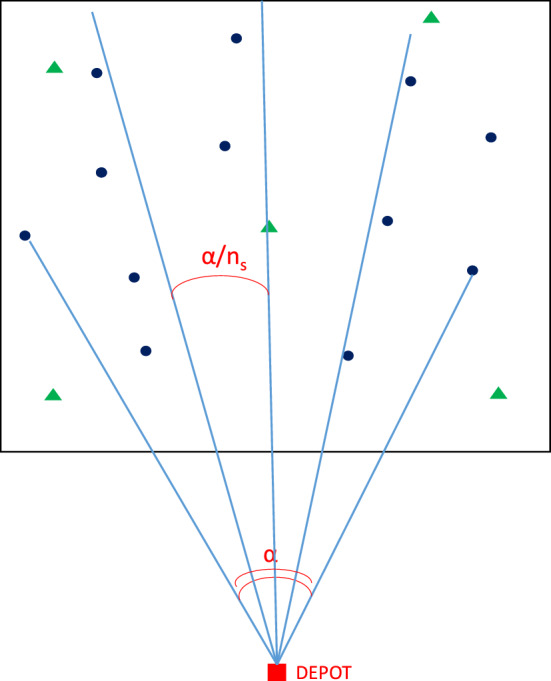
2D corridors representation

**Fig. 5:**
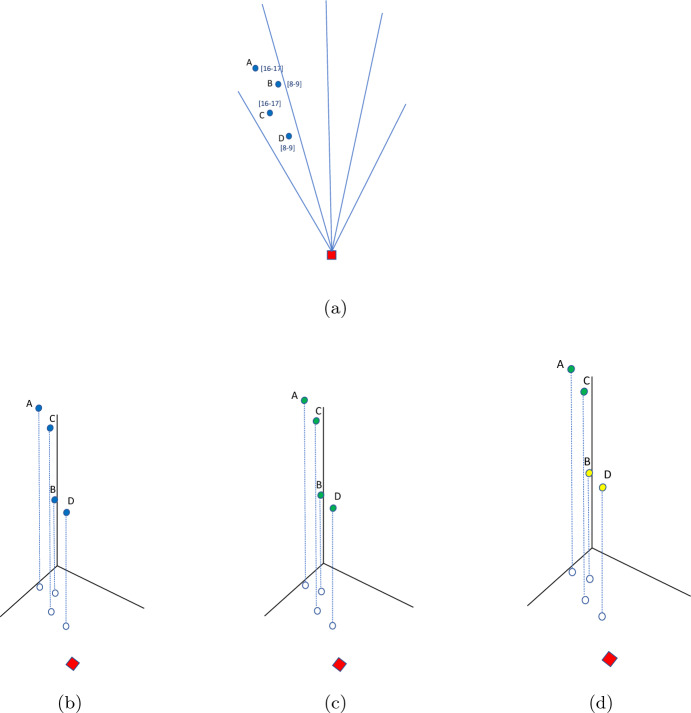
An example of bundle generation with 3D corridors and 2D corridors. Subfigure (**a**) reports customers’ locations and TWs, (**b**) depicts customers’ 3D representation, (**c**) shows the bundles of customers generated by exploiting a 2D corridors approach, and in (**d**) bundles obtained by exploiting a 2D corridors approach, can be seen. The requests belonging to the same bundle are depicted in the same color (Color figure online)

Notet that the 2D corridors approach proposed in Mancini and Gansterer (2022a) only rely on customers’ locations and not on their TWs. Corridors are circular sectors lying on the horizontal plane, as depicted in Fig. 4. In this case, all the customers belonging to the same sector, identified as the set $S_{n_s}$, are considered as a single bundle (if the vehicle capacity allows for it) despite their TWs. In Fig. 5 we depict an example in which four customers are considered (A,B,C,D). All of them belong to the same 2D corridor, but they have completely different TWs. In fact, B and D must be served in the early morning ([8–9]), while A and C in the late afternoon ([16–17]). Following a 2D corridors approach, all the customers would be bundled together, but the resulting bundle would not result attractive. In fact, a driver should serve D and B in the early morning and then come back to C and wait more than six hours to serve C and D. This bundle would require a long detour in terms of distance and a very long temporal detour, which make it very unattractive. Conversely, the 3D corridors approach would split in two attractive bundles, (B,D) and (A,C), which would result very attractive, requiring a short detour both in terms of distance and time. In fact, an OD operating in the early morning, can serve the first bundle (B,D) while (A,C) can be easily served by a driver operating in the afternoon.

Resuming the method requires the following five parameters as input: the vehicle speed *v*, the origin of the first plane (*t*0), the thickness of the slice, *T* (from which the origin of the second plane *t*1 can be derived), and the number of slices to be created ($n_s$). The larger the value of *T*, the larger the time flexibility of the driver and the number of feasible bundles that can be created. But, on the other side, larger values of *T* can lead to feasible bundles with long waiting times required to meet all the TWs. Such bundles can be of limited attractiveness for the ODs. Moreover, more flexibility implies a larger number of feasible bundles, which may increase the complexity of the problem to be solved. Conversely, small values of *T* could allow a limited number of bundles to be generated and potential loss of some profitable bundles, resulting in a poor global solution.

As for the number of slices ($n_s$), the smaller this value, the larger the allowed detour and number of feasible bundles created. It is important to analyze different values of $n_s$, as smaller values can identify large bundles of customers, who require a relatively long detour, but might be very attractive because of the large compensation received for serving several customers. Conversely, larger values, allow to create bundles that contain only a few customers (or even single-customer bundles), which can be attractive also for drivers with a low flexibility. Both types of bundles are needed for a successful auction and effective global delivery plan. All bundles generated with different combinations of parameters (see Sect. 6) are inserted in the auction pool, excluding duplicates. It is worth noting that since given a fixed combination of values of ($n_S$,$t_0$, and $t_1$), the slices created are disjoint among each other, also the bundles generated so far are disjoint. But, since we run the algorithm for different combination of those parameters, we might have a set of partial overlapping bundles. This holds also for bundles generated with the Clustering 3D approach, in which, given a fixed number of clusters $N_n$, the bundles generated are disjoint, but bundles generated with different value of $N_n$, can be partially overlapping.

### Bidding system

The bundles that are offered and the underlying OD bidding system is a complex and critical issue for the whole system. In the seminal paper on the VRP with ODs, Archetti et al. (2016), the authors do not contemplate a bidding phase but assign a fixed compensation for each delivery, regardless of the detour for the OD. This compensation scheme, despite being simple to implement in practice, could be considered unfair from the drivers’ perspective. Although two ODs may incur completely different extra mileages to serve the same bundles, they would receive exactly the same compensation. The authors stick to their assumption by arguing that to pay a compensation proportional to the actual detour required, it would be necessary to know the home locations of all the ODs. This could expose the system to strategic behavior and cheating, as ODs could declare that they live very far away from the customers’ area to receive a higher compensation. Moreover, the authors do not contemplate the possibility that ODs may reject a task if it does not match their preferences or if the compensation offered for the required detour is too low.

Differently from Archetti et al. (2016), in Dahle et al. (2019) the authors consider that the ODs may reject an offer proposed by the company if the compensation paid is too low. The authors do not explicitly model the possibility of ODs refusing tasks, but they overcome this issue by imposing a minimum compensation threshold for each OD.

In Ausseil et al. (2022), the authors do not implement an auction-based system but consider a centralized platform that provides personalized request menus for each driver, which depends on the drivers’ final destination. The drivers can select one of the proposed requests or can decline all the offers if they do not match their interest. Even if the authors do not consider an actual bidding system, they take into account driver preferences by considering the possibility of less attractive offers being rejected.

Hammami et al. (2022) and Triki et al. (2014) investigate a bidding system for combinatorial auctions in transportation, but in both papers, no bundles are generated by the company. Instead, an authority offers all the single requests for which drivers can make combined bids or for subsets of multiple requests. Hence, both the problems are very different from ours.

A bundling and bidding system is used in Mancini and Gansterer (2022a), where the company offers a set of bundles in the auction pool, and drivers submit their bids. This is the first attempt to actually give freedom to the drivers to implement their own bidding strategy, based on their flexibility and willingness to work. The company receives the bids from the drivers without knowing how prices are computed. However, to model realistic behavior on the part of drivers, an automatic bidding system that simulates rational OD behavior (based on locations, flexibility, and willingness to work) is considered.

In our study, We adopt the same auction-based system used by Mancini and Gansterer (2022a), but we implement a different automatic bidding system where drivers’ availability period during the day and their tolerance into accepting waiting times at customers are also taken into account. Both of these features are because of the TWs being demanded by customers.

Each OD $\omega$ is fully characterized by the following parameters: (i) an availability period; (ii) *flexibility*, which represents the maximum acceptable detour, computed as the difference between the shortest path from the depot to the OD’s final destination, visiting all the customers in the bundle without violating their TWs and the direct path from the depot to the OD’s final destination; (iii) *willingness to work* ($\phi _\omega$), where $\phi _\omega =1$ describes a truthful behavior, i.e., the ODs’ bids reflect exactly the actual detour implied. In case of a lower willingness (i.e., $\phi _\omega >1$), the bid prices are increased, as the ODs agree to do a delivery only if they find it very convenient. Values smaller than 1 ($\phi _\omega <1$) indicate that the driver is really willing to work and, therefore, reduces the bid price to become more competitive in the market, and by doing so, have a greater chance of winning the bundle. It should be noted that a value of $\phi _\omega =1$ does not reflect a zero-profit bid (i.e., it does not merely cover the expenses of the driver) but one that generates a profit being considered standard in this type of job. Therefore, even for values of $\phi _\omega <1$, it is still possible to achieve a significant profit.

The value of a bid *k* is calculated as the detour length, $\delta _k$, expressed as the detour needed by OD, $o_k$, to serve customers belonging to bundle $\tau _k$, multiplied by a unitary distance cost, $c^u$, with a fixed service cost, $c^f$ added to it for each customer belonging to the bundle and a waiting cost, $w_k$, representing the waiting time that ODs may face when serving all the customers in the bundle, multiplied by an individual-willingness-to-wait-factor for each driver ($\psi _{o_k}$). We consider a proxy value for $w_k$, which is determined as follows: $w_k=e_{max}-l_{min}$, where $e_{max}$ is the latest TW-starting time among all the customers in the bundle $\tau _k$, while $l_{min}$ is the earliest TW-ending time in this bundle. The sum of these three costs (distance, service, and waiting cost) is then multiplied by the willingness to work parameter ($\phi _{o_k}$). Hence, the bid price $b_k$ is formulated as follows:

26 $$\begin{aligned} b_k=(c^u\delta _k+c^f|\tau _k|+\psi _{o_k} w_k)\phi _{o_k} \end{aligned}$$

The detour $\delta _k$ is obtained by solving a modified version of the Open Travelling Salesman Problem with Time Windows (OTSP-TW), considering the depot as the starting node, the customers belonging to the bundle as the further required nodes, and the OD’s final destination as the final node. Additionally, it is assumed that the starting time from the depot and the arrival time at the final destination lie within the ODs’ availability period. If this problem does not admit a feasible solution, we consider the bundle incompatible with the driver, who therefore, does not submit any bid for this bundle.

The flexibility level does not influence the bidding price but indirectly impacts the bidding process, for we assume that an OD places a bid for a bundle only if the required detour $\delta _k$ is lower than the maximum value accepted ($\delta ^{MAX}_{o_k}$).

Summing up, we consider a compensation scheme based on the detour required, without forcing ODs to reveal their home locations to the company. It must be noted that our auction system receives bids as input data, so it works independently from the bidding strategy considered. Hence, every strategy, rational or irrational, can be used in the system.

However, to have realistic bids and draw managerial insights, we use a mechanism simulating ODs’ real behavior.

## Computational study

The aim of our computational experiments is threefold and as follows: (i) For the newly proposed Corridors-3D approach, we analyze the impact of the thickness of 3D slices on the quality of the generated bundles; (ii) We compare the Corridors-3D approach with the more straightforward and classical Clustering-3D approach; (iii) We analyze the improvement achievable by opting for a spatial-temporal approach to generate bundles, using only spatial information.

### Experimental setting

We construct 6 sets of instances, in turn composed of 10 sub-instances each. All the sets are adapted from those provided by Mancini and Gansterer (2022a). The first 3 of them, namely 20*STW*, 20*MTW*, and 20*LTW*, include 10 ODs and 20 customers and are characterized by small, medium, and large TWs, respectively. The other 3 sets (40*STW*, 40*MTW*, 40*LTW*) have 20 ODs and 40 customers with small, medium, and large TWs. In all *STW* instances, we consider 1-hour-wide, non-overlapping TWs. Medium-sized TWs have a width of 2 h and are partially overlapping (e.g., [8–10], [9–11], and [10–12]). In all the *LTW* instances, we consider only 3 TWs ([8–12], [12–16], and [16–20]), each with a 4 h-width. As mentioned in the previous sections, ODs are characterized by flexibility and willingness, where flexibility indicates the accepted deviation from the shortest path, and willingness is reflected in an OD’s bidding behavior. Since onboarding ODs in a TWs-based delivery planning is more complex because of lower compatibility of customers and the possible extra detour and extra waiting times imposed by TWs, we believe that only very motivated and flexible drivers can be helpful in this context. Therefore, we set the value of flexibility and willingness to 3 and 0.6, respectively, which represents a high value for both of the features. As we are interested in showing the maximum potential benefit achievable by onboarding ODs, we consider that all of them are available for the whole day. This does not mean that an in-store customer has to wait to meet customer TWs but that ODs can flexibly plan the time of their visit at the store and to adapt to the deliveries they are offered on that day. An analysis of the impact of different combinations of flexibility and willingness to work have been carried out in Mancini and Gansterer (2022a). The authors state that there is no significant impact of these two parameters on the performances of the bundles generation approaches (clustering and corridors). For this reason we do not report a similar analysis but we focus our attention to ODs with high levels of flexibility and willingness, which are the ones that can provide a higher benefit for the company.

Fixed and mileage costs, $c_u$ and $c_f$, are set to 2 and 1.5, respectively, as in Mancini and Gansterer (2022a), whereas the waiting cost, $\Psi _{o_k}$, is considered homogeneous for all the drivers and is set at 2. The number of available owned fleet vehicles is 5 for all the instances. The cost of doing a delivery, using a taxi service is considered as 100.

For the Corridors-3D approach, we assume *t*1 to be constant, but we perform several runs of the algorithm, letting *t*0 vary in the range $[e_0;e_1-1]$, *T* in [1 and 4] and $n_s$ in [10, 20, 30, and 40]. Different values of *t*0 represent different time slots of a day when the driver can start.

The instances are publicly available in Mancini and Gansterer (2022b). For all experiments, a machine equipped with a 11th Gen Intel Core i7–1185G7 with 32 GB of RAM is used. The mathematical model is run under Xpress 8.13 with standard settings and a time limit of 3600 s. The optimality gap tolerance was set to 10^−5^, which is the common default value.

### Impact of slice thickness in 3D corridors

To evaluate the impact of the 3D slice thickness, we compare 4 different values (1, 2, 3, and 4 h) for all the 6 sets of instances. As mentioned above, the thickness of a slice might strongly influence the bundle generation, as, for instance, very thin slices may find only a limited number of feasible bundles. It could even happen that only single-customer bundles are generated, basically due to incompatible TWs, with a potential loss of the profit achievable by onboarding ODs. On the contrary, very thick slices would include bundles, involving a very large number of customers, and potentially, long waiting times and detours may be required to serve all them, which make these bundles unattractive for ODs. This could yield to a situation in which the number of bundles offered is large but the number of bids submitted is very limited, and this would reduce the benefit of onboarding ODs as well. Even if bids are submitted, these might be very high, which makes it more convenient for the company to use the owned fleet. If ODs are limited in use or not profitable, this could yield to situations in which it is not possible to serve all the customers and/or taxi services may need to be booked for some of the deliveries, which would entail relatively high costs for the company.

What emerges from this analysis is that the slice thickness does not have a strong impact on the costs. The number of bundles generated is quite limited in all the cases, but their attractiveness and profitability is very high. This can be evinced by the following two facts: first, despite a small number of bundles, the number of bids received is quite high (3–4 bids per bundle in the small instances, 7 in the large ones), as reported in Table 2; second, the percentage of customers served by ODs is quite high (50–55% for the small instances and 45–50% for the large ones), which means that the bids are profitable for the company. Moreover, it is worth noting that when dealing with small and medium TWs, the number of bundles generated slightly increase with the thickness of the slice. Conversely, when TWs are large, the number of bundles decreases. This could seem counterintuitive, but can be explained by observing the average size of the bundles (i.e., the number of customers included in a bundle), which is larger. In fact, larger slices can contain several customers, yielding to the creation of fewer bundles but with a larger average size. This effect only applies when the TWs are large enough to guarantee a high compatibility among customers. Instead, when TWs are smaller, it is difficult to obtain bundles with a large number of customers due to the impossibility to respect customers TWs.

**Fig. 6:**
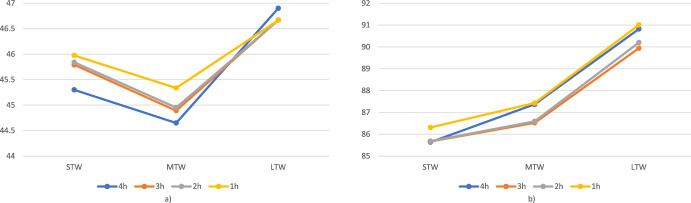
Best found solutions’ objective function for different slice thickness values, for small (**a**) and large (**b**) instances

Globally, the thickness of three hours seems to yield the best results, as reported in Fig. 6. Hence, we keep this value for the benchmarking against other bundle generation approaches. Further, observing Tables 3, 4, 5, 6, 7, 8 in the Appendix, we can note that computational times rapidly increase with the growth of instance size. Note that this increment is devoted to the higher difficulty in solving the optimization model (1)–(20), as both the bundling and the bidding phase still require very short times. Conversely, the length of TWs does not significantly impact the computational times.

**Table 2:** Number of bundles (#Bu) and of bids (#Bi) generated by the 3D corridors approach with different values of thickness

	1 h		2 h		3 h		4 h	
	#Bu	#Bi	#Bu	#Bi	#Bu	#Bi	#Bu	#Bi
20STW	44.2	163.4	47	172.9	50.3	179.6	54.6	190.6
20MTW	45.4	170.5	50	180.8	51.1	183.1	54.3	190.7
20LTW	37.5	135.8	37.5	135.8	37.5	135.8	36	131.8
40STW	110.8	709.5	124.2	779.3	128.8	810.2	133.8	836
40MTW	112.8	717.4	124.7	777.8	133.6	828	135.3	841.3
40LTW	85.1	572.9	85.1	571.8	86.1	577.6	77.5	523.1

### Corridors-3D versus clustering-3D

In this subsection, we compare the performance of the two 3D-bundling approaches, namely Corridors-3D (with a thickness of three hours) against the 3D-Clustering one. We provide data related to (i) applying only the Corridors-3D approach, (ii) applying only the Clustering-3D approach, and (iii) applying a combination of both of them.

**Fig. 7:**
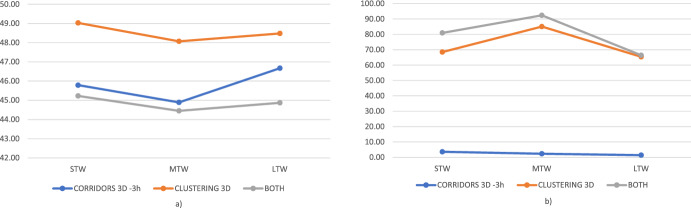
Comparison between the Corridors-3D and Corridors-2D approach on the small (**a**) and large (**b**) instances

As can be observed from Fig. 7, Corridors-3D outperforms Clustering-3D in all the instances in solution quality and particularly in regards to computational times. Nevertheless, mixing up the two approaches and considering all the bundles generated so far (excluding duplicated ones) would improve solution quality slightly (while computational times are still high). This is so because some of the cluster-generated bundles are profitable. It can be seen that this holds particularly for the instances with large TWs, and it can be explained by the fact that since only 3 TWs with high probability are available, namely ([8–12], [12–16], and [16–20]), clustered customers are also near in time. However, the results show that solution quality decreases if these bundles are not combined with bundles generated by Corridors-3D. Nevertheless, given the very high computational times required for running the clustering-3D method, we believe that the advantage of including Cluster-3D bundles is negligible. Moreover, clustering approaches are not suitable to handle the large instances, as computational times grow quickly, and it would take several hours to generate the bundles, as already pointed out by Mancini and Gansterer (2022a). For all the above explained reasons, the Corridors-3D approach is strongly preferable over the Clustering-3D method.

### Corridors-3D versus corridors-2D

**Fig. 8:**
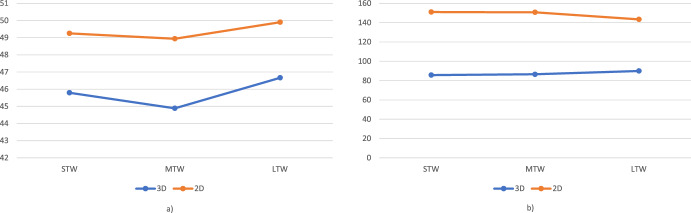
Comparison between the Corridors-3D and Corridors-2D approach on the small (**a**) and large (**b**) instances

The scope of this analysis is to compare the Corridors-3D approach, which takes into account the spatial-temporal customer representation, with a Corridors-2D approach, which only considers the spatial location of customers. The latter approach is also used in Mancini and Gansterer (2022a). In this case, the corridors lay on a 2D plane and are, therefore, defined by circular sectors instead of by 3D slices. Average results are reported in Fig. 8, while detailed results can be found in Tables 12 and 13 in the Appendix.

On the small instances, the 3D approach outperforms the 2D approach, generating more attractive bundles in considerably shorter amount of time. The number of ODs involved decreases if the 2D approach is applied, as well as the percentage of customers served by ODs, which goes from 55% (with the 3D approach) to less than 40%.

On large instances, the superiority of the 3D approach is even more evident. The costs almost double when the 2D approach is used. This can be explained by the fact that the bundles generated are of less attractiveness for ODs, so several customers have to be served either by owned fleet or by hiring a taxi service.

Resuming, we can conclude that the Corridors-3D approach significantly outperforms the other approaches both in terms of solution quality and computational times.

## Conclusions and future developments

In this paper we introduced the vehicle routing problem with occasional drivers and time windows (VRP-OD-TW), where customer orders can be fulfilled by ODs, by the fleet owned by a company, or by means of direct shipment, using a taxi service. The latter option is costly and inconvenient for the company, but it ensures that all the orders can be fulfilled on time. To address the cost problem, we proposed an auction-based system, in which the company generates potentially attractive bundles for the ODs. The latter place bids for the bundles they are interested to serve. Once the company receives all the bids, it determines, based on an optimization model, which bids to accept. Finally, a routing plan for the owned fleet and the assignment to the taxi service is generated.

To generate bundles, we proposed an innovative customer representation in a 3D space, wherein the first two dimensions deal with the spatial location of customers, while the third represents their delivery TWs. The bundles are generated based on the creation of 3D corridors, named slices. We analyzed the impact of the thickness of the slices on the performance of the approach. We also compare this approach with a more classical one, in which we applied a clustering technique, aiming at minimizing intra-cluster distance in the 3D spatial-temporal environment. Finally, we compared the Corridors-3D approach with the Corridors-2D approach (in which the temporal aspect of the problem is neglected). The results showed a clear dominance of the newly proposed Corridors-3D approach on the other two methods in terms of both solution quality and computational times.

Future research can address the adoption of the spatial-temporal representation to create attractive bundles of customers for other problems arising in logistics such as, for instance, pickup and delivery problems with ODs or auction-based collaborative routing problems.

## Appendix

We report detailed results from all the analyses conducted in this study. In Tables 3, 4, and 5, we show the averaged results obtained by adopting different slice thicknesses (1–4 h) on the small instances with short, medium, and long TWs, respectively. We report the average values for the objective function (OF), lower bounds (LBs), run times in seconds for bundling (TIME_BUND), bidding (TIME_BID), model solved (TIME_SOL), and total run time (TIME_TOT). The last four rows give the average numbers of bundles, bids, ODs, and customers served by ODs.

**Table 3:** Comparison of results of the small instances with short TWs for different slice thicknesses

	1 h	2 h	3 h	4 h
OF	45.98	45.84	45.79	45.30
LB	45.97	45.84	45.79	45.30
TIME_BUND	0.05	0.08	0.13	0.17
TIME_BID	0.20	0.31	0.41	0.52
TIME_SOL	2.53	2.51	3.07	2.40
TIME_TOT	2.78	2.90	3.62	3.09
# BUNDLES	44.2	47.00	50.30	54.60
# BIDS	163.40	172.90	179.60	190.60
# OD	7.30	7.20	7.00	7.00
# SERVED by ODs	11.40	11.30	11.30	11.90

**Table 4:** Comparison of results of the small instances with medium TWs for different slice thicknesses

	1 h	2 h	3 h	4 h
OF	45.34	44.95	44.89	44.65
LB	45.34	44.95	44.89	44.65
TIME_BUND	0.09	0.15	0.23	0.30
TIME_BID	0.23	0.37	0.41	0.49
TIME_SOL	2.15	1.72	1.69	1.62
TIME_TOT	2.46	2.24	2.33	2.41
# BUNDLES	45.40	50.00	51.10	54.30
# BIDS	170.50	180.80	183.10	190.70
# OD	6.20	6.10	6.40	6.60
# SERVED by ODs	10.30	10.40	10.80	11.30

**Table 5:** Comparison of results of the small instances with long TWs for different slice thicknesses

	1 h	2 h	3 h	4 h
OF	46.67	46.67	46.67	46.90
LB	46.67	46.67	46.67	46.90
TIME_BUND	0.04	0.06	0.08	0.11
TIME_BID	0.08	0.08	0.08	0.08
TIME_SOL	1.29	1.27	1.25	1.65
TIME_TOT	1.41	1.41	1.41	1.86
# BUNDLES	37.50	37.50	37.50	36.00
# BIDS	135.80	135.80	135.80	131.80
# OD	6.40	6.40	6.40	6.60
# SERVED by ODs	10.70	10.70	10.70	10.70

The same information is reported in Tables 6, 7, 8 for the large instances with short, medium, and long TWs.

**Table 6:** Comparison of results of the large instances with short TWs for different slice thicknesses

	1 h	2 h	3 h	4 h
OF	86.31	85.68	85.67	85.64
LB	86.02	85.49	85.25	85.37
TIME_BUND	0.30	0.56	0.93	1.82
TIME_BID	1.21	2.12	4.21	15.07
TIME_SOL	1,301.82	1,249.33	1,402.40	1,321.72
TIME_TOT	1,303.33	1,252.01	1,407.54	1,338.61
# BUNDLES	110.80	124.20	128.80	133.80
# BIDS	709.50	779.30	810.20	836.00
# OD	12.40	11.70	12.60	12.00
# SERVED by ODs	21.30	20.80	22.10	21.50

**Table 7:** Comparison of results of the large instances with medium TWs for different slice thicknesses

	1 h	2 h	3 h	4 h
OF	87.43	86.59	86.52	87.37
LB	86.21	85.78	85.65	86.53
TIME_BUND	0.59	0.61	1.05	1.56
TIME_BID	4.79	1.84	2.65	3.18
TIME_SOL	2320.86	1822.75	2095.16	2642.16
TIME_TOT	2326.24	1825.20	2098.86	2646.90
# BUNDLES	112.80	124.70	133.60	135.30
# BIDS	717.40	777.80	828.00	841.30
# OD	10.90	11.40	11.90	11.20
# SERVED by ODs	19.20	19.90	20.60	19.40

**Table 8:** Comparison of results of the large instances with long TWs for different slice thicknesses

	1 h	2 h	3 h	4 h
OF	91.01	90.20	89.94	90.82
LB	87.94	89.03	89.32	90.07
TIME_BUND	10.38	3.26	1.14	1.50
TIME_BID	18.42	5.19	1.01	0.98
TIME_SOL	3385.40	1800.18	1973.19	1966.32
TIME_TOT	3414.20	1808.63	1975.33	1968.80
# BUNDLES	85.10	85.10	86.10	77.50
# BIDS	572.90	571.80	577.60	523.10
# OD	11.10	11.20	11.10	11.40
# SERVED by ODs	18.00	17.80	18.00	17.90

The comparison of the Corridors-3D with the Clustering-3D on the small instances, with short, medium, and long TWs is reported in Tables 9, 10, and 11, respectively. We report the average values for the objective function (OF), lower bounds (LBs), run times (in seconds) for bundling, bidding, model solved, and total run time. The last four rows give the average numbers of bundles, bids, ODs, and customers served by ODs.

**Table 9:** Comparison of Corridors-3D (three-hour slices) against Clustering-3D and against a combination of both the approaches on the small instances with short TWs

	Corridors-3D	Clustering-3D	Both
OF	45.79	49.02	45.23
LB	45.79	49.02	45.23
TIME_BUND	0.13	60.49	59.42
TIME_BID	0.41	0.95	18.21
TIME_SOL	3.07	7.11	3.35
TIME_TOT	3.62	68.55	80.98
# BUNDLES	50.30	40.30	72.80
# BIDS	179.6	154.60	249.10
# OD	7.00	6.90	7.00
# SERVED by ODs	11.30	8.50	11.90

**Table 10:** Comparison of Corridors-3D (three-hour slices) against Clustering-3D and against a combination of both the approaches on the small instances with medium TWs

	Corridors-3D	Clustering-3D	Both
OF	44.89	48.07	44.45
LB	44.89	48.07	44.45
TIME_BUND	0.23	65.94	70.86
TIME_BID	0.41	14.29	19.19
TIME_SOL	1.69	4.84	2.35
TIME_TOT	2.33	85.07	92.39
# BUNDLES	51.10	42.80	76.60
# BIDS	183.10	161.30	261.50
# OD	6.40	7.00	6.70
# SERVED by ODs	10.80	8.90	11.80

**Table 11:** Comparison of Corridors-3D (three-hour slices) against Clustering-3D and against a combination of both the approaches on the small instances with long TWs

	Corridors-3D	Clustering-3D	Both
OF	46.67	48.48	44.87
LB	46.67	48.48	44.87
TIME_BUND	0.08	62.75	63.75
TIME_BID	0.08	0.87	1.47
TIME_SOL	1.25	1.80	1.24
TIME_TOT	1.41	65.42	66.45
# BUNDLES	37.50	40.50	59.10
# BIDS	135.80	152.80	204.80
# OD	6.40	6.70	7.20
# SERVED by ODs	10.70	9.10	12.10

Finally, in Tables 12 and 13 we provide a detailed comparison between the Corridors 3D and the Corridors 2D approach on small and large instances, respectively. We report the average values for the objective function (OF), lower bounds (LBs), run times (in seconds) for bundling, bidding, model solved, and total run time. The last four rows give the average numbers of bundles, bids, ODs, and customers served by ODs.

**Table 12:** Comparison of the Corridors-3D against the Corridors-2D approach on small instances

	Short TW		Medium TW		Long TW	
	3D	2D	3D	2D	3D	2D
OF	45.79	49.25	44.89	48.94	46.67	49.90
LB	45.79	49.25	44.89	48.94	46.67	49.90
TIME_BUND	0.13	0.94	0.23	1.12	0.08	0.99
TIME_BID	0.41	0.11	0.41	0.28	0.08	0.11
TIME_SOL	3.07	3.17	1.69	6.30	1.25	1.35
TIME_TOT	3.62	4.21	2.33	7.70	1.41	2.45
# BUNDLES	50.30	23.70	51.10	22.70	37.50	23.10
# BIDS	179.60	81.10	183.10	81.40	135.80	80.90
# OD	7.00	5.00	6.40	4.40	6.40	5.00
# SERVED by ODS	11.30	7.90	10.80	7.60	10.70	8.30

**Table 13:** Comparison of the Corridors-3D against the Corridors-2D approach on large instances

	Short TW		Medium TW		Long TW	
	3D	2D	3D	2D	3D	2D
OF	85.67	151.03	86.52	150.76	89.94	143.42
LB	85.25	150.21	85.65	149.28	89.32	143.39
TIME_BUND	0.93	0.62	1.05	13.57	1.14	13.86
TIME_BID	4.21	0.66	2.65	1.90	1.00	3.21
TIME_SOL	1402.40	2352.65	2095.16	2985.83	1973.19	576.72
TIME_TOT	1407.54	2353.93	2098.85	3001.30	1975.33	593.78
# BUNDLES	128.80	40.70	133.60	42.70	86.10	42.70
# BIDS	810.2	284.50	828.00	294.60	577.60	293.60
# OD	12.60	8.30	11.90	8.40	11.10	8.70
# SERVED by ODS	22.10	15.30	20.60	15.20	18.00	15.30
